# Haemodialysis-Induced Stress Influences Ocular Microcirculation

**DOI:** 10.3390/biomedicines14020454

**Published:** 2026-02-18

**Authors:** Joanna Roskal-Wałek, Sylwia Terpiłowska, Joanna Gołębiewska, Jerzy Mackiewicz, Kamila Bołtuć-Dziugieł, Agnieszka Bociek, Paweł Wałek, Michał Biskup, Dominik Odrobina, Andrzej Jaroszyński

**Affiliations:** 1 Ophthalmology Clinic, Voivodeship Regional Hospital, 25-736 Kielce, Poland; michal.biskup@onet.pl; 2 Collegium Medicum, Institute of Medical Sciences, Jan Kochanowski University, 25-516 Kielce, Poland; sylwia.terpilowska@ujk.edu.pl (S.T.); kbdziugiel@gmail.com (K.B.-D.); lekagnieszkabociek@gmail.com (A.B.); pawel.walek@o2.pl (P.W.); magdale_5@hotmail.com (D.O.); jaroszynskiaj@interia.pl (A.J.); 3 Department of Ophthalmology, Military Institute of Aviation Medicine, 01-755 Warsaw, Poland; 4 Medical Faculty, Lazarski University, 02-662 Warsaw, Poland; 5 Department of Vitreoretinal Surgery, Medical University of Lublin, 20-059 Lublin, Poland; jerzy.mackiewicz@umlub.pl; 6 1st Clinic of Cardiology and Electrotherapy, Swietokrzyskie Cardiology Centre, 25-736 Kielce, Poland; 7 Ophthalmology Clinic Boni Fratres Lodziensis, 93-357 Łódź, Poland

**Keywords:** haemodialysis, oxidative stress, asymmetric dimethylarginine, endothelin-1, malondialdehyde, optical coherence tomography angiography

## Abstract

**Background:** Haemodialysis (HD) superimposes additional circulatory stress on the microvasculature, leading to endothelial dysfunction, which plays a key role in the development of haemodialysis-associated multiorgan dysfunction. This study was undertaken to evaluate the effect of a single HD session on retinal and choroidal microcirculation, using optical coherence tomography angiography (OCTA) in relation to changes in the blood levels of selected biochemical modulators of endothelial function. **Methods:** The vessel density (VD) of 35 patients was evaluated before and after a single HD session, using OCTA in the superficial capillary plexus (SCP), deep capillary plexus (DCP) and choriocapillaris (CC). Retinal thickness (RT) and choroidal thickness (CT) were also assessed. Asymmetric dimethylarginine (ADMA), endothelin-1 (ET-1) and malondialdehyde (MDA) levels, oxidative stress (OS) status and systemic parameters were assessed before and after a single HD session. The correlation between changes in these parameters and changes in selected OCTA parameters was tested. **Results:** A single HD session resulted in a significant increase in RT and a decrease in CT. In addition to increased oxidative and osmotic stress resulting from a significant reduction in plasma osmolality, the HD session was associated with a significant increase in ET-1 levels and a decrease in ADMA levels. These biochemical changes correlated with changes in RT and CT, as well as with changes in VD in the retinal capillary plexuses and the CC. Increased ET-1 levels and decreased plasma osmolality were identified as predictors of RT increase, whereas increased MDA levels corrected serum creatinine-predicted CT reduction. **Conclusions:** Changes in ADMA and ET-1 and OS, as well as osmotic stress induced by a single HD session, affect the eye microcirculation and morphology of the retina and choroid. OCTA examination is a promising method for assessing microcirculation in HD patients.

## 1. Introduction

Chronic kidney disease (CKD) has become a major global health challenge, affecting almost 850 million people worldwide. Haemodialysis (HD) is the most common life-sustaining form of kidney replacement therapy in the world [1,2]. Although HD saves patients’ lives, the HD process itself exposes patients to multiple episodes of circulatory stress, exacerbating and being aggravated by microvascular endothelial dysfunction, which plays a key role in the development of HD-associated cardiovascular harm [3,4]. Most of the endothelial surface area is part of the microcirculation; therefore, assessing the impact of HD on microcirculation is crucial to fully understand the consequences of HD treatment [5].

HD has been shown to result in ischaemic insults affecting the myocardium, renal and brain [3,4]. Moreover, similar blood flow perturbations occur in other vascular beds [3,4,5,6]. Despite the variety of methods available to study microcirculation, these techniques have largely not been incorporated into routine clinical practice [7]. Optical coherence tomography angiography (OCTA) provides a quick, non-invasive, and repeatable evaluation of retinal and choroidal microcirculation, offering valuable insight into how ocular microcirculation responds to a single HD session [8]. The effect of HD on retinal and choroidal microcirculation is gaining attention. To date, there are only a few studies that have assessed retinal and choroidal microcirculation changes due to HD sessions, but these results vary [9,10,11,12,13,14,15]. Whether the HD procedure itself contributes to ocular microcirculation dysfunction is still incompletely investigated.

HD induced changes in the levels of vasoactive factors such as plasma asymmetrical dimethylarginine (ADMA), an endogenous inhibitor of nitric oxide synthase, and plasma endothelin-1 (ET-1), a powerful vasoconstrictor. Moreover, HD can result in changes in levels of oxidative stress (OS) markers, such as malondialdehyde (MDA) [16,17,18,19,20,21]. All these substances modulate endothelial function and may influence ocular microcirculation [15,21,22,23].

To the best of our knowledge, the link between changes in OS and vasoactive factors on eye microcirculation due to a single HD session has not yet been investigated. Therefore, this study aimed to investigate the effect of a single HD session on the retinal and choroidal microcirculation in relation to changes in parameters representing OS, as well as vasoactive factors.

## 2. Material and Methods

This prospective observational study was carried out from March to May 2022 at the Provincial General Hospital in Kielce, in collaboration with the Ophthalmology Clinic and the Nephrology Clinic. The study protocol received approval from the Bioethics Committee of the Jan Kochanowski University in Kielce (6/2022) and was conducted in accordance with the Declaration of Helsinki. All participants provided written informed consent.

We prospectively enrolled 48 patients with stage 5 chronic kidney disease (ESRD) undergoing HD for at least three months. Participants were ≥18 years of age and were classified according to the aetiology of ESRD as being hypertensive, diabetic, glomerulonephritis, polycystic kidney disease, vasculitis, other, or unknown when documentation was insufficient or multiple causes were suspected. The inclusion criteria required a best corrected visual acuity (BCVA) that was better than 6/60 and clear ocular media. The exclusion criteria included spherical equivalent refractive errors that were greater than +3.0 D or more than −5.0 D, an axial length (AXL) under 21 mm or over 26 mm, glaucoma, infection, and any retinal or choroidal pathology that could affect the tissue structure, such as uveitis, retinal vascular disease, macular degeneration, macular oedema, macular hole, or epiretinal membrane. Also excluded were patients with a history of eye injury or ocular procedures, including intravitreal injections, laser therapy, or eye surgery, except for uncomplicated cataract surgery performed at least 12 months prior to the study enrolment. OCT images of insufficient quality (below 60, according to the manufacturer), whether due to eye movements or media opacities, were also excluded. Additional exclusion criteria included atrial fibrillation, acute coronary syndrome or cerebral stroke within the previous three months, and severe infection or exacerbation of a chronic illness within one month before the study.

All participants underwent three 4 h HD sessions weekly, with a dialysate flow rate of at least 500 mL/min and a blood flow rate of at least 300 mL/min. Patients were examined only during the short interval between dialysis sessions, and all measurements were performed in the dialysis centre building. To avoid diurnal variations in the choriocapillaris flow, only individuals receiving morning HD sessions were included.

### 2.1. Ophthalmic Parameters

Each participant underwent a complete ophthalmic examination 30 min before HD (8 a.m.) and 15 min after HD. Spherical equivalent was measured using an automated refractometer (Righton Speedy-K2, UKON Corporation, Tokyo, Japan). AXL was measured using optical biometry (Tomey OA-2000 Optical Biometer, Tomey Corporation, Aichi, Japan).

OCTA acquisition was performed after administering 1% tropicamide eye drops for pupil dilatation. OCTA is a novel non-invasive imaging technology that uses intrinsic blood flow motion contrast to produce angiographic data across the retina and choroid [24]. All scans were obtained using swept-source OCT-A (DRI-OCT Triton SS-OCT Angio, Topcon Inc., Tokyo, Japan). The scanning protocol consisted of a 3 × 3 mm field centred on the fovea. For analysis, one eye per patient was selected based on superior OCT-A image quality, with all scans being required to meet a minimum quality score of 60%.

Macular vessel density (VD) is one of the quantitative metrics that can be obtained from OCTA images and refers to the proportion of the macula with blood flow compared to the total macular area [25]. Vessel density was calculated automatically by the software as the percentage area occupied by blood vessels in the three vascular plexuses: superficial capillary plexus (SCP) from 2.6 µm below the internal limiting membrane (ILM) to 15.6 µm below the interface of the inner plexiform layer and inner nuclear layer (IPL/INL), deep capillary plexus (DCP) from 15.6 µm below the IPL/INL to 70.2 µm below the IPL/INL, and choriocapillaris (CC) from Bruch’s membrane (BM) to 10.4 µm below BM, using the ETDRS grid subfields to define the areas of interest. The central VD corresponded to the central field, and the entire 3 × 3 mm macular scan is referred to as the total VD.

Choroidal thickness (CT) and retinal thickness (RT) were measured automatically in nine ETDRS subfields, using a 7 × 7 mm 3D macular scan. Total CT and total RT were defined as the average thickness of all nine subfields, while central CT and central RT referred to measurements in the central ETDRS subfield.

### 2.2. Systemic Parameters

Body weight was recorded, and systolic (SBP) and diastolic blood pressure (DBP) were measured before and after the HD session. Mean arterial pressure (MAP) was calculated as DBP + 1/3 × (SBP − DBP). Blood pressure measurements were performed using the AtCor SphygmoCor Xcel device (AtCor Medical, Sydney, Australia).

The ultrafiltration volume, indicating the amount of fluid removed during HD, was recorded after the procedure.

### 2.3. Biochemical Parameters

(1) **Serum urea and creatinine**

These parameters were estimated using ready-to-use ALPHA Diagnostics kits, using a Mindray 200 E biochemical analyser.

(2) **CRP**

This parameter was estimated using commercial kits.

(3) **ET-1**

The concentration of ET-1 was measured using a ready-to-use test kit, according to the original manufacturer’s instructions (Cloud-Clone Corp, Katy, TX, USA). The detection range of a test was 6.17–500 pg/mL.

(4) **IL-6**

The IL-6 concentration was measured using a ready-to-use test kit, according to the original manufacturer’s instructions (Diaclone, Besançon, France). The detection range of a test was 6.25–200 pg/mL and the sensitivity was 2 pg/mL.

(5) **ADMA**

This parameter was measured photometrically using a ready-to-use test kit, following the original manufacturer’s instructions (Immundiagnostik AG, Bensheim, Germany). The optical density (OD) in all tests was measured with the use of a Synergy HTX multi-mode reader (Biotek, Winooski, VT, USA).

(6) **MDA**

Serum MDA was measured using a ready-to-use test kit, according to the original manufacturer’s instructions (Cloud-Clone Corp, USA).

The MDA levels were corrected for serum creatinine and expressed as µg/mg of creatinine. Taking into consideration the clearance of MDA during dialysis, the ratio of MDA and creatinine was likely to give a better picture of the MDA levels during dialysis [17].

(7) **Plasma osmolality**

This parameter was calculated using an Osmometer 800 CLG (Trident-Med s.c. Warsaw, Poland).

(8) **Serum total oxidant status/total oxidant capacity (TOS/TOC) and total antioxidant status/total antioxidant capacity (TAS/TAC).**

These parameters were measured photometrically using ready-to-use test kits, following the original manufacturer’s instructions (KC5100 and KC5200, respectively; Immundiagnostik AG, Germany). The TAS/TAC kit evaluates antioxidant capacity by measuring the reaction between sample antioxidants and a known amount of exogenously added hydrogen peroxide (H_2_O_2_), while the TOS/TOC kit quantifies total lipid peroxides.

### 2.4. Statistical Analysis

The distribution of quantitative variables was checked using the Shapiro–Wilk test, and then means, standard deviations, medians, and interquartile ranges were calculated in the patient groups before and after the HD session. Student’s *t*-test or the Wilcoxon signed-rank test were used to compare the measurements of the overall patient group at two time points—before and after the HD session—depending on the distribution of characteristics. Correlations between measurements were calculated using Pearson’s or Spearman’s correlation tests. Multiple linear regression was used to determine the effect of variable increments (MDA corrected for serum creatinine; plasma osmolality on the change in total CT and ET-1; plasma osmolality on the change in central RT). All model assumptions were verified. All statistical analyses were performed using the STATISTICA 13.3 software (TIBCO Software Inc., Tulsa, OK, USA).

## 3. Results

### 3.1. Baseline Characteristics

A total of 48 patients were examined, of which 35 patients (17 males, 18 females; age, 32–83 years; mean age 61.14 ± 12.01) met the inclusion criteria, and 35 eyes were enrolled in the study. The aetiology of ESRD included diabetes mellitus (n = 8), glomerulonephritis (n = 7), hypertension (n = 6), polycystic kidney disease (n = 4), vasculitis (n = 2), other (n = 3), and unknown (n = 5). The baseline characteristics of the group are presented in Table 1.

### 3.2. Effect of Haemodialysis Session on Retinal and Choroidal Thickness

The mean total and central RT significantly increased after the HD session, contrary to the mean central and total CT, which significantly decreased after the HD in all subfields. The detailed changes in all subfields of the above-mentioned parameter are presented in Table 2.

### 3.3. Effect of Haemodialysis on Vessel Density

Vessel density in the SCP, DCP and CC did not change significantly in any of the subfields. The trend towards an increase in the total VD in DCP is noteworthy; however, this result did not reach significance. Detailed changes in the perfused vessel density of the macula after HD are listed in Table 3.

### 3.4. Effects of Haemodialysis Session on Biochemical Parameters

Following the HD session, significant changes occurred in biochemical parameters. After the HD session, CRP and TOS/TOC increased significantly, as did MDA corrected for serum creatinine (*p* < 0.001). The HD session also resulted in a significant increase in ET-1 (*p* < 0.0004) and a decrease in ADMA levels (*p* < 0.001) and TAS/TAC (*p* < 0.0136). The plasma osmolality changed during HD from 321.12 ± 9.81 to 302.79 ± 7.66 mOsm/kg (*p* < 0.0001). Detailed changes in the biochemical parameters are presented in Table 4.

### 3.5. Correlations Between Changes Induced by a Single Haemodialysis Session in Biochemical Parameters and Changes in RT, CT and VD

Changes in the ET-1 levels were positively correlated with changes in the central and total RT and were negatively correlated with changes in total SCP VD.

Changes in the ADMA levels were positively correlated with changes in the total DCP VD.

Changes in the MDA levels corrected for serum creatinine were negatively correlated with changes in the CT.

Changes in the TOS/TOC were negatively correlated with changes in the total SCP, central, and total CC VD.

Changes in the TAS/TAC were negatively correlated with changes in the total SCP and total DCP VD.

Changes in the plasma osmolality were positively correlated with changes in the central and total CT and were negatively correlated with changes in the central and total RT. In addition, changes in the plasma osmolality were positively correlated with changes in the VD in the total DCP and total CC. Details of the relationship between changes in the biochemical parameters and changes in VD and retinal and choroid thickness are presented in Table 5.

### 3.6. Correlations Between Changes Induced by a Single Haemodialysis Session in Systemic Factors and Changes in RT, CT and VD

The changes in SBP, DBP, and MAP, as well as the change in the body weight, did not show a correlation with the changes in RT, CT or VD in SCP, DCP, or CC. Details of the relationship between changes in SBP, DBP, and MAP and body weight and changes in RT, CT, and VD in SCP, DCP, and CC are presented in Table 6.

### 3.7. Predictors of Changes in Retinal and Choroidal Thickness

An increase of 1 unit in ΔET-1 is associated with a 0.17% increase in central RT, assuming that the plasma osmolality remains constant. Conversely, a decrease of 1 unit in plasma osmolality is associated with a 0.2% increase in RT, assuming that the ΔET-1 remains constant. Both changes in plasma osmolality and ΔET-1 are significant and together account for 32.71% of the total variation in RT. The results of the multiple regression analysis identifying independent predictors of an RT increase are presented in Table 7.

Of the two assessed predictors of CT—namely, changes in MDA corrected for serum creatinine and plasma osmolality—only an increase in MDA corrected for serum creatinine significantly predicts a decrease in CT. A one-unit increase in MDA corrected for serum creatinine is associated with a 0.15% decrease in CT, assuming plasma osmolality remains constant. Together, changes in the plasma osmolality and serum creatinine–corrected MDA account for 20.12% of the total variation in CT. The results of the multiple regression analysis identifying independent predictors of CT decrease are presented in Table 8.

## 4. Discussion

Our study generated three major findings. First, we demonstrated that a single HD session resulted in significant changes in the morphology of the retina and choroid (increasing and decreasing their thickness, respectively). Second, we found that a single HD session resulted in an increased OS status, as well as increased osmotic stress, due to a significant decrease in plasma osmolality. The HD session also resulted in significant changes in the levels of vasoactive factors, such as the ET-1 and ADMA concentrations. Third, we demonstrated that these biochemical changes correlated with changes in RT and CT, as well as changes in VD in retinal plexuses and in CC. The predictors of an RT increase were both an increase in ET-1 and a decrease in plasma osmolality, while the decrease in CT was associated with an increase in the MDA concentration corrected for serum creatinine.

Our results, indicating a significant increase in RT and a decrease in CT, are consistent with the findings of Wang et al. [26]. In the studies by Coppolino et al. and Shin et al., CT also decreased, but RT did not change significantly [10,12]. In the study by Zegrari et al., the subfoveal CT significantly decreased after the HD session, which was accompanied by a significant increase in non-perfusion in the choriocapillaris [14]. Given the exceptionally high blood-flow-to-tissue ratio in the choroid, this structure may be particularly sensitive to HD-related microcirculatory changes [8,27]. Recently, Lin et al. found an increase in RT after the HD session and suggested that an increase in VD in DCP may be involved in this change [15]. In our study, we only found a tendency to increase VD in total DCP; however, this change did not reach significance. VD in SCP and CC did not show significant changes. In contrast, in the study by Coppolino et al., significant reductions were observed in the 6 × 6 whole SCP VD and 6 × 6 foveal DCP VD. Several other studies show no changes in retinal plexuses after HD sessions [10,14].

Due to the lack of neuronal innervation, the regulation of retinal blood flow relies mainly on local regulatory mechanisms, including oxygen, carbon dioxide, nitric oxide (NO), ET-1, angiotensin II and other mediators [28]. Several studies have shown improved retinal microcirculatory function during single HD sessions with a retinal vessel diameter increase [29,30,31]. Recently, increased VD in DCP was shown by Lin et al. It was assumed that the reduction in ADMA levels during HD resulted in increased NO availability and vasodilation [15]. However, no study to date has assessed the effect of ADMA changes on VD following a HD session.

In our study, we have shown that a HD session produces a significant decrease in ADMA, which is in agreement with the results of some other works [16]. Moreover, the change in ADMA levels showed a positive correlation with the changes in total VD in DCP, which shows a tendency to increase, which seems to confirm previous assumptions.

Although there was a decrease in ADMA during HD, we also observed an increase in ET-1 levels, the most potent known vasoconstrictor [28]. Enhanced levels of ET-1 may contribute to endothelial dysfunction through inhibitory effects on NO production [32].

In our study, increased levels of ET-1 correlate positively with central and total RT increase and negatively with the total change in SCP VD.

The lack of significant changes in the retinal and choroidal VD as a result of increased NO availability due to decreased ADMA may also be due to NO inactivation as a consequence of increased production of reactive oxygen species (ROS) [18]. Increased ROS production impairs the balance of NO metabolism and affects the responsiveness of vascular endothelial and smooth muscle cells to physiological stimuli, such as shear stress and vasoactive substances, which may ultimately be associated with blood flow changes in the heart, brain, and eye [22].

Similarly to other studies, we showed a significant increase in OS levels after a single HD session [17,33]. The significant increase in post-HD MDA corrected for serum creatinine was an indicator of the presence of OS during the dialysis session, which, in our study, is confirmed by an increase in TOS/TOC. Moreover, during the HD session, antioxidants are lost through the neutralisation of ROS and passage across the dialyser membrane, further intensifying OS [34]. In our study, the TAS/TAC was also significantly decreased. Because of its permanent exposure to light and its composition of several susceptible tissues with high metabolic activities, low antioxidant enzyme levels and an abundant content of oxidisable structures, the visual system is very vulnerable to OS [23]. Increased OS was shown to be involved in the pathophysiology of various ocular diseases. Vascular dysfunction is recognised to be part of the pathogenesis of several retinal pathologies, such as diabetic retinopathy, retinal vessel occlusion, age-related macular degeneration, and glaucoma [23].

In our study, the increase in OS showed a negative correlation with the change in VD in SCP and CC.

However, it is worth bearing in mind that ROS can have opposing influences on the tone of a vessel, depending on the condition and type of the vessel. At resting potential, ROS provoke dilation; however, in precontracted vessels, they induce further vasoconstriction [35].

It is also worth noting that haemodialysed patients showed worsening vascular reactivity. It is possible that the endothelial damage observed in ESRD patients is irreversible and cannot be significantly affected by HD, which could also explain the lack of significant changes in VD in our study [9].

Interestingly, we found that an increase in MDA corrected for serum creatinine correlated negatively with a decrease in CT. Previous studies have indicated that OS may affect CT and lead to decreased CC VD [36,37]. However, this is the first study to indicate that increased MDA corrected for serum creatinine due to HD session affects choroidal morphology.

It is not clear why increased TOS/TOC did not show an association with CT that was comparable to MDA. This result may be due to the small study group size or possibly due to the fact that MDA, as a small molecule, can easily pass through choroidal vessels, which show multiple fenestrations, making them highly permeable to proteins and several macromolecules [38]. The effect of increased levels of MDA corrected for serum creatinine on CT and the lack of such an effect on RT may be due to the fact that choroidal circulation is mainly controlled by extrinsic autonomic innervation [8,27].

We can speculate that an increased OS may lead to dysfunction of the autonomic nervous system (ANS) [39], which manifests as sympathetic hyperactivity and/or reduced parasympathetic activity and may be caused by stressors associated with HD [40]. Therefore, the CT decrease may result from the stronger constriction of the vascular smooth muscles and non-vascular smooth muscles in the choroid due to the activation of the sympathetic ANS [8,31]. Dysfunction of the ANS may also be caused by other stressors related to the HD process.

In addition to the effect of OS, our study showed that a single HD session also resulted in osmotic stress in the form of a significant reduction in plasma osmolality. Oxidative and osmotic stress differ significantly while also displaying overlapping responses [41].

Bołtuć-Dziugieł et al. showed that a key factor influencing OS during the HD session could be changes in plasma osmolality [42].

In a multivariate regression analysis, we demonstrated that a decrease in plasma osmolality, along with ET-1, predicts an increase in RT, while an increase in MDA corrected for creatinine is a predictor of CT decrease.

Osmotic stress leads to an efflux or influx of water from or into the cell: hyperosmotic stress causes shrinking and hypoosmotic stress causes swelling [41]. Chen et al. assumed that HD reduces the plasma crystal osmotic pressure in such a way that the liquid flows into the retinal layers according to the concentration gradient, thickening the retina and leading to oedema [43]. Our findings support these assumptions. The cellular responses to this type of stress deal with the activity of water channels (aquaporins-AQP) and electrolyte transporters, the accumulation of osmolytes, and the protection of proteins and subcellular structures [41]. We hypothesise that AQP type and expression may contribute to the RT increases observed in this and previous studies; however, this requires further investigation [13].

Recurrent changes following HD sessions, such as increased OS and osmotic stress accompanied by increased ET-1, may be responsible for the subsequent decline in the CT, RT and VD values observed in patients undergoing HD [44,45]. However, this assumption requires verification in further studies. Moreover, changes in CT due to HD may lead to retinal pigment epithelium dysfunction, the main function of which is the metabolism of peroxidised polyunsaturated fatty acids (PUFA) from photoreceptor outer segments (POS). MDA is a product of PUFA peroxidation, and POS have the highest concentration of PUFA in the body, which makes them very susceptible to peroxidation [46].

Our study has several limitations. The relatively small sample size and the lack of follow-up assessments after subsequent HD sessions limit the generalisability of the findings and preclude reliable subgroup analyses, including sex-stratified comparisons and analyses across ESRD aetiologies. Autonomic nervous system function was not evaluated, although it represents a promising area for future research. Potential confounding factors such as changes in blood gases, pH, or medication use were not accounted for, which may have influenced the measured parameters. The 3 × 3 mm scanning protocol was chosen for superior image quality; however, wider scans might provide additional insights into HD-induced microcirculatory changes. Finally, given the preliminary nature of this study, the proposed mechanisms underlying changes in RT and CT in relation to biochemical parameter alterations should be interpreted with caution and confirmed in larger, adequately powered cohorts.

This is the first study to comprehensively examine HD-related changes in biochemical endothelial markers alongside retinal and choroidal microcirculatory responses. We also evaluated systemic parameter changes and their effects on ocular circulation. OCTA metrics were obtained using automated software, eliminating observer-related measurement error. All assessments were performed immediately before and after HD, within a single centre, minimising the variability related to timing and external factors.

## 5. Conclusions

The present study findings suggest that a single HD session results in significant changes in RT and CT. Significant changes in ET-1, ADMA, OS and plasma osmolality following a single HD session affect retinal and choroidal microcirculation and morphology. OCTA examination is a promising method for assessing microcirculation in HD patients.

## Figures and Tables

**Table 1:** Baseline characteristics of the study group.

Patients’ Characteristic	Before HD	After HD	*p*-Value
Age (years) Mean (SD)	61.14 ± 12.01		
Gender Females (%) Males (%)	18 (51.43%) 17 (48.57%)		
Duration of HD (years) Mean (SD)	4.85 ± 3.65		
Ultrafiltration volume (l) Mean (SD)	2.19 ± 1.03		
kt/v Mean (SD)	1.6 ± 0.31		
Spherical equivalent, (D) Mean (SD)	0.24 ± 1.47		
Axial length, (mm) Mean (SD)	22.97 ± 0.79		
Body weight (kg)	77.83 ± 18.64	75.64 ± 18.21	**<0.0001 ^T^**
SBP (mmHg)	141.54 ± 27.63	137.65 ± 28.66	0.4638 ^T^
DBP (mmHg)	77.63 ± 13.7	78.41 ± 14.18	0.7154 ^T^
MAP (mmHg)	98.93 ± 17.42	98.16 ± 18.09	0.9715 ^T^

**Abbreviations**: DBP, diastolic blood pressure; HD, haemodialysis; MAP, mean arterial pressure; SBP, systolic blood pressure; and SD, standard deviation. ^T^ Student’s *t* test for dependent variables. Statistically significant difference (*p* < 0.05).

**Table 2:** Effects of haemodialysis session on retinal and choroidal thickness.

	RT			CT		
	Before HD	After HD	*p*-Value	Before HD	After HD	*p*-Value
Total	269.11; 23.00 ^B^	272.00; 21.22 ^B^	0.0015 ^W^	220.09 ± 65.40 ^A^	206.63 ± 65.60 ^A^	0.0001 ^T^
Central	234.23 ± 27.36 ^A^	238.14 ± 25.73 ^A^	0.0282 ^T^	230.46 ± 71.04 ^A^	215.83 ± 68.68 ^A^	0.0001 ^T^
OS	261.00; 22.00 ^B^	262.00; 24.00 ^B^	0.0064 ^W^	234.89 ± 70.68 ^A^	223.57 ± 73.62 ^A^	0.0046 ^T^
ON	270.00; 33.00 ^B^	272.00; 21.00 ^B^	0.0052 ^W^	182.29 ± 72.97 ^A^	171.06 ± 74.79 ^A^	0.0001 ^T^
OI	251.00; 23.00 ^B^	251.00; 22.00 ^B^	0.2823 ^W^	202.00; 120.00 ^B^	197.00;121.00 ^B^	0.0001 ^W^
OT	246.00; 21.00 ^B^	244.00; 23.00 ^B^	0.0118 ^W^	208.89 ± 64.22 ^A^	196.51 ± 64.15 ^A^	0.0001 ^T^
IS	299.00; 26.00 ^B^	300.00; 23.00 ^B^	0.0099 ^W^	239.23 ± 70.54 ^A^	227.26 ± 68.40 ^A^	0.0013 ^T^
IN	296.00; 30.00 ^B^	303.00; 27.00 ^B^	0.0008 ^W^	220.14 ± 75.98 ^A^	207.20 ± 73.94 ^A^	0.0011 ^T^
II	295.00; 24.00 ^B^	302.00; 24.00 ^B^	0.0018 ^W^	228.14 ± 72.06 ^A^	215.54 ± 71.45 ^A^	0.0001 ^T^
IT	285.00; 19.00 ^B^	285.00; 16.00 ^B^	0.0163 ^W^	224.74 ± 71.83 ^A^	207.14 ± 68.28 ^A^	0.0001 ^T^

**Abbreviations:** CT, choroidal thickness; RT, retinal thickness; HD, haemodialysis; OS, outer superior; ON: outer nasal; OI, outer inferior; OT, outer temporal; IS, inner superior; IN, inner nasal; II, inner inferior; and IT, inner temporal. Statistically significant difference (*p* < 0.05). ^T^ Student’s *t*-test for dependent variables; ^W^ Wilcoxon signed-rank test; ^A^ M—mean ± SD—standard deviation; ^B^ Me—median; and Iqr—interquartile range. Data are presented as mean ± SD for normally distributed variables and median (IQR) for non-normally distributed variables.

**Table 3:** Effects of haemodialysis on vessel density.

	VD SCP			VD DCP			VD CC		
	Before HD	After HD	*p*-Value	Before HD	After HD	*p*-Value	Before HD	After HD	*p*-Value
Total	40.38;2.80 ^B^	41.18;3.05 ^B^	0.2870 ^W^	39.98;3.38 ^B^	40.21;3.06 ^B^	0.0553 ^W^	52.63;2.01 ^B^	52.70;1.60 ^B^	0.1197 ^W^
Central	20.40 ± 4.11 ^A^	20.83 ± 4.28 ^A^	0.3600 ^T^	17.89 ± 4.29 ^A^	18.81 ± 7.99 ^A^	0.1923 ^T^	50.87;5.54 ^B^	52.56;4.80 ^B^	0.1277 ^W^
Superior	45.71;4.95 ^B^	45.46;4.89 ^B^	0.9543 ^W^	45.53;4.97 ^B^	46.85;6.16 ^B^	0.5337 ^W^	51.99;4.85 ^B^	51.59;2.57 ^B^	0.4711 ^W^
Nasal	44.87;3.23 ^B^	45.18;3.18 ^B^	0.9673 ^W^	45.54 ± 4.27 ^A^	45.11 ± 3.66 ^A^	0.4301 ^T^	53.53 ± 2.27 ^A^	53.06 ± 1.63 ^A^	0.1760 ^T^
Inferior	45.15 ± 3.67 ^A^	45.83 ± 4.55 ^A^	0.3426 ^T^	45.02;5.42 ^B^	46.26;5.91 ^B^	0.4463 ^W^	52.03;3.41 ^B^	51.25;5.65 ^B^	0.3217 ^W^
Temporal	45.37 ± 3.75 ^A^	45.60 ± 3.51 ^A^	0.7090 ^T^	43.66;4.69 ^B^	43.84;4.66 ^B^	0.2689 ^W^	53.69;2.91 ^B^	53.50;3.74 ^B^	0.7494 ^W^

**Abbreviations:** CC, choriocapillaris; DCP, deep capillary plexus; HD, haemodialysis; SCP, superficial capillary plexus; and VD, vessel density. ^T^ Student’s *t*-test for dependent variables; ^W^ Wilcoxon signed-rank test ^A^ M—mean; SD—standard deviation; ^B^ Me—median; and Iqr—interquartile range. Data are presented as mean ± SD for normally distributed variables and median (IQR) for non-normally distributed variables. Statistically significant difference (*p* < 0.05).

**Table 4:** Effects of haemodialysis session on biochemical parameters.

Parameter	Overall (n = 35)		
	Before HD	After HD	*p*-Value
Plasma osmolality (mOsm/kg)	321.12 ± 9.81 ^A^	302.79 ± 7.66 ^A^	0.0001 ^W^
Creatinine (mg/dL)	7.81 ± 2.41 ^A^	2.65 ± 0.92 ^A^	<0.0001 ^T^
Urea (mg/dL)	122.80 ± 30.79 ^A^	36.07 ± 11.17 ^A^	<0.0001 ^W^
CRP (mg/dL)	5.79;4.58 ^B^	6.22;5.15 ^B^	0.0001 ^W^
IL-6 (pg/mL)	90.50 ± 23.48 ^A^	90.07 ± 23.86 ^A^	0.0697 ^T^
ADMA (mmlo/L)	0.54 ± 0.14 ^A^	0.34 ± 0.16 ^A^	0.0001 ^W^
ET-1 (pg/mL)	10.17 ± 6.64 ^A^	15.05 ± 6.78 ^A^	0.0004 ^W^
MDA corrected for serum creatinine (µg/mg creatinine)	27.11 ± 13.29 ^A^	77.02 ± 37.52 ^A^	0.0001 ^W^
TOS/TOC (mmol/L H_2_O_2_)	78.19;309.71 ^B^	250.88;376.89 ^B^	0.0093 ^W^
TAS/TAC (mmol/L)	286.47;88.95 ^B^	219.57;109.30 ^B^	0.0136 ^W^

**Abbreviations:** ADMA, Asymmetric dimethylarginine; CRP, C-reactive protein; ET-1, Endothelin-1; IL-6, interleukin-6; MDA, malondialdehyde; TAC, total antioxidant capacity; TAS, total antioxidant status; TOC, total oxidant capacity; and TOS, total oxidant status. ^T^ Student test for dependent variables; ^W^ Wilcoxon signed-rank test; ^A^ M—mean; SD—standard deviation; ^B^ Me—median; Iqr—interquartile range. Data are presented as mean ± SD for normally distributed variables and median (IQR) for non-normally distributed variables.

**Table 5:** Correlations between changes induced by single haemodialysis session in biochemical parameters and changes in RT, CT and VD.

Variables	Δ ADMA (n = 33)		Δ ET-1 (n = 31)		Δ MDA/Krea (n = 33)		ΔTOS/TOC (n = 32)		ΔTAS/TAC (n = 32)		Δ Plasma Osmolality (n = 34)	
	Rho ^S^/r ^P^	*p*	Rho ^S^	*p*	Rho	*p*	Rho ^S^/r ^P^	*p*	Rho ^S^	*p*	Rho ^S^/r ^P^	*p*
Δ central RT	0.08	0.644 ^S^	0.46	0.010 ^S^	−0.05	0.771 ^S^	0.14	0.455 ^S^	−0.05	0.767 ^S^	−0.35	0.045 ^S^
Δ total RT	−0.04	0.823 ^S^	0.54	0.002 ^S^	−0.02	0.926 ^S^	0.10	0.587 ^S^	0.811	0.54 ^S^	−0.37	0.034 ^S^
Δ central CT	0.04	0.827 ^P^	−0.12	0.505 ^S^	−0.27	−1.547 ^S^	−0.09	0.617 ^P^	0.06	0.732 ^S^	0.35	0.047 ^P^
Δ total CT	−0.05	0.772 ^P^	−0.11	0.558 ^S^	−0.41	0.019 ^S^	−0.14	0.437 ^P^	−0.04	0.834 ^S^	0.37	0.038 ^P^
Δ central SCP VD	0.25	0.169 ^P^	−0.31	0.093 ^S^	−0.14	0.426 ^S^	−0.33	0.065 ^P^	−0.24	0.191 ^S^	0.04	0.836 ^P^
Δ total SCP VD	0.33	0.058 ^S^	−0.43	0.017 ^S^	−0.02	0.912 ^S^	−0.53	0.002 ^S^	−0.53	0.002 ^S^	0.26	0.136 ^S^
Δ central DCP VD	0.17	0.344 ^P^	−0.19	0.294 ^S^	−0.01	0.966 ^S^	−0.20	0.278 ^P^	−0.11	0.550 ^S^	0.05	0.766 ^P^
Δ total DCP VD	0.34	0.049 ^S^	−0.34	0.058 ^S^	0.00	0.985 ^S^	−0.30	0.091 ^S^	−0.40	0.023 ^S^	0.47	0.005 ^S^
Δ central CC VD	−0.02	0.915 ^S^	−0.12	0.521 ^S^	−0.04	0.819 ^S^	−0.40	0.024 ^S^	−0.35	0.051 ^S^	0.30	0.086 ^S^
Δ total CC VD	−0.07	0.701 ^P^	−0.08	0.651 ^S^	0.00	0.987 ^S^	−0.41	0.019 ^P^	−0.29	0.102 ^S^	0.62	0.001 ^P^

**Abbreviations:** ADMA, Asymmetric dimethylarginine; CC, choriocapillaris; DCP, deep capillary plexus; ET-1, Endothelin-1; MDA, malondialdehyde; SCP, superficial capillary plexus; TAC, total antioxidant capacity; TAS, total antioxidant status; TOC, total oxidant capacity; TOS, total oxidant status; VD, vessel density ^S^—Spearman’s rank correlation; ^P^—Pearson’s correlation.

**Table 6:** Correlations between changes induced by a single haemodialysis session in systemic factors and changes in RT, CT and VD.

Variables	Δ SBP		Δ DBP		Δ MAP		Δ Body Weight	
	Rho/r	*p*	Rho/r	*p*	Rho/r	*p*	Rho/r	*p*
Δ central RT	−0.14	0.426 ^S^	−0.20	0.248 ^S^	−0.18	0.317 ^S^	−0.05	0.797 ^S^
Δ Total RT	−0.13	0.469 ^S^	−0.24	0.167 ^S^	−0.19	0.281 ^S^	−0.17	0.342 ^S^
Δ Central CT	0.24	0.186 ^P^	0.18	0.312 ^P^	0.22	0.224 ^P^	−0.09	0.605 ^P^
Δ Total CT	0.27	0.133 ^P^	0.32	0.070 ^P^	0.31	0.083 ^P^	0.09	0.614 ^P^
Δ Central SCP VD	0.06	0.727 ^P^	0.05	0.775 ^P^	−0.02	0.923 ^P^	−0.09	0.619 ^P^
Δ Total SCP VD	0.07	0.680 ^S^	0.09	0.597 ^S^	0.08	0.733 ^S^	−0.02	0.910 ^S^
Δ Central DCP VD	0.28	0.112 ^P^	0.20	0.254 ^P^	0.07	0.763 ^P^	−0.14	0.434 ^P^
Δ Total DCP VD	0.15	0.398 ^S^	0.22	0.215 ^S^	0.19	0.424 ^S^	−0.23	0.188 ^S^
Δ Central CC VD	−0.14	0.426 ^S^	−0.06	0.733 ^S^	−0.07	0.763 ^S^	−0.14	0.432 ^S^
Δ Total CC VD	0.12	0.515 ^P^	0.20	0.252 ^P^	0.02	0.934 ^P^	−0.16	0.366 ^P^

**Abbreviations:** CC, choriocapillaris; CT, choroidal thickness; DBP, diastolic blood pressure; DCP, deep capillary plexus; MAP, mean arterial pressure; SBP, systolic blood pressure; SCP, superficial capillary plexus; RT, retinal thickness; and VD, vessel density. ^S^—Spearman’s rank correlation; ^P^—Pearson’s correlation.

**Table 7:** Predictors of RT increase.

Predictors	β	95% CI	SE	*p*
Δ ET-1	0.17	0.01; 0.32	0.08	0.035
Δ plasma osmolarity	−0.20	−0.33; −0.07	0.06	0.004
R^2^ = 0.3271; *p* = 0.001				

**Abbreviations:** ET-1, Endothelin-1; **CI—confidence interval; SE—standard error**. Statistically significant difference (*p* < 0.05).

**Table 8:** Predictors of CT decrease.

Predictors	β	95% CI	SE	*p*
Δ MDA corrected for serum creatinine	−0.15	−0.28; −0.01	0.07	0.034
Δ Plasma osmolarity	0.32	−0.10; −0.73	0.20	0.129
R^2^ = 0.2012; *p* = 0.013				

**Abbreviations: MDA, malondialdehyde.** Statistically significant difference (*p* < 0.05).

## Data Availability

The data presented in this study are available on reasonable request from the corresponding author.
